# The Role of Capoeira in Improving Motor and Social Skills in Children with Autism

**DOI:** 10.3390/children12101305

**Published:** 2025-09-27

**Authors:** Roei Lev-Ari, Meir Lotan, Liat Korn

**Affiliations:** 1 Department of Health Systems Management, Faculty of Health Sciences, Ariel University, Ariel 40700, Israel; liatk@ariel.ac.il; 2 Department of Physiotherapy, Faculty of Health Sciences, Ariel University, Ariel 40700, Israel; meirlo@ariel.ac.il

**Keywords:** autism, capoeira, inclusion, motor skills, social skills development, sensory regulation, adapted physical activity

## Abstract

**Highlights:**

**What are the main findings?**

- Capoeira training improved motor skills such as coordination, balance, and body awareness in children with autism.
- Participation fostered social communication and peer interaction, enhancing children’s confidence and engagement.

**What is the implication of the main finding?**

- Capoeira can serve as a culturally rich and inclusive physical activity program supporting both motor and social development.
- The findings suggest practical pathways for integrating children with autism into mixed-group physical education and community programs.

**Abstract:**

Background: Children with Autism often face motor, sensory and communicational challenges that can hinder their participation in meaningful physical and social activities. This study explores the potential of Capoeira to support their development across these domains. Methods: This qualitative pilot study used semi-structured interviews with parents of children aged 7–15 with Autism Spectrum Disorder (ASD) who participated in group Capoeira programs. Data were analyzed through content categorization, leading to the development of thematic constructs. Results: Three central domains emerged regarding the perceived impact of Capoeira training on children with Autism: 1. Improvements in areas commonly affected in Autism, including sensory and auditory regulation, motor coordination, bodily awareness, compliance, and social communication. 2. Increased motivation, independence, sense of belonging and integration into mixed peer groups of typically developing (TD) children and children with ASD. 3. Broader developmental gains were also reported, such as increased self-confidence, initiative, awareness of others, and transfer of skills beyond the training context. Conclusions: The findings suggest that Capoeira may serve as an effective integrated intervention model, supporting physical and social development in children with ASD. Capoeira was reported to be associated with improved coordination, balance, body awareness, and gains in nonverbal interaction and social engagement, all within a collaborative, non-competitive framework. Future studies should explore the short and long-term impact of such interventions through quantitative outcome measures, as well as clarify the mechanisms that promote successful integration.

## 1. Introduction

Autism Spectrum Disorder (ASD) is a neurodevelopmental condition. It is characterized by challenges in social communication, the presence of repetitive behaviors, and restricted interests [1]. Official definitions emphasize impairments in social-emotional reciprocity, nonverbal communicative behaviors, such as body language and eye contact, and difficulties in interpreting social cues [2,3]. Additional characteristics of ASD may include a pronounced insistence on routines, repetitive rituals, and atypical sensory sensitivities, which can involve unusual responses to sounds, tastes, or textures [4,5,6]. Such symptoms can contribute to significant challenges in personal, social, and occupational functioning. A fundamental aspect of ASD is the difficulty in establishing and maintaining friendships. These emotionally significant relationships necessitate intimacy, stability, and reciprocity, all of which depend on well-developed social-cognitive abilities [7,8]. Children diagnosed with ASD frequently encounter obstacles in expressing positive emotions, sharing experiences, maintaining eye contact, and engaging in cooperative interactions [9]. Consequently, their friendships are often fewer in number and inferior in quality compared to those of typically developing peers, which may adversely impact their present and future social and emotional well-being [4,5,10].

### 1.1. Motor Difficulties in Children with ASD

Approximately 87% of children with ASD struggle with age-appropriate motor tasks, particularly in areas such as ball skills, balance, and visuomotor coordination [11,12,13,14]. These challenges are clinically significant, affecting not only motor skills, but also social engagement, information processing, and executive functioning [15,16,17]. Moreover, the severity of the motor issues was found to correlate with the severity of core elements of ASD [18,19,20], while the presence of motor difficulties was found to negatively influence the advancement of children with ASD in regard to their core difficulties [20]. Research indicates that motor interventions, such as physical activity, swimming, therapeutic horseback riding, and hydrotherapy, can lead to improvements of 16% to 43% in skills like throwing, catching, manual dexterity, and balance [21,22,23]. Furthermore, enhancements in motor functioning have been linked to better executive functions, communication, and social behaviors, as well as increased serotonin uptake [24,25,26,27,28,29]. Despite the recognized importance of physical activity, children with ASD participate in such activities significantly less often than their peers. A study in the United States found that only 17% of boys with ASD engaged in daily physical activity, compared to 33% in the general population [30].

### 1.2. Gross Motor Skills and Social Competence in ASD

A meta-analysis incorporating 21 studies has established a significant relationship between gross motor impairments and deficiencies in social skills among individuals with ASD [17]. These motor impairments are posited to hinder participation in social activities, as evidenced by observations that children with poor gross motor skills exhibit lower social integration and diminished peer popularity [31]. Additionally, a systematic review and meta-analysis of 16 quantitative studies, comprising both randomized controlled trials (RCTs) and quasi-experimental designs, revealed that motor-based interventions, including adapted basketball, karate, therapeutic horseback riding, and swimming, resulted in notable improvements of up to 77% in social communication skills in children with ASD [32]. Evaluations of these improvements utilized standardized assessment instruments such as the Social Responsiveness Scale (SRS-2). Furthermore, notable advancements were observed in emotional regulation, self-awareness, and overall mood, alongside reductions in repetitive behaviors and rigidity, as assessed by tools like the Repetitive Behavior Scale-Revised (RBS-R). An RCT focusing on children with ASD indicated that participation in a 12-week running program markedly enhanced emotional regulation and led to a decrease in externalizing behaviors and overall behavioral issues, as measured by the Emotional Regulation Checklist (ERC) and the Child Behavior Checklist (CBCL). These findings may also imply indirect enhancements in social communication skills through the facilitation of improved emotional and behavioral functioning [33]. Moreover, gross motor skills have been linked to social-cognitive competencies, including executive functions (EF) and theory of mind (ToM), both of which are essential for effective social interactions [34]. These results emphasize the necessity for interventions that integrate motor, cognitive, and social-cognitive elements to foster optimal social functioning in children with ASD [35]. Previous literature has identified martial arts as efficacious interventions for bolstering executive functioning and social competencies in this population [36,37]. Within this paradigm, Capoeira, a motor-social activity that synthesizes physical, emotional, and cognitive dimensions emerges as a particularly promising intervention warranting further exploration.

### 1.3. Capoeira

Capoeira is a distinctive Afro-Brazilian martial art that emphasizes cooperation among participants. The Capoeira game is characterized by dynamic interaction, where practitioners move in sync, incorporating kicks, evasions, dance-like movements, and acrobatic elements. Unlike many other martial arts that focus on defeating an opponent, Capoeira aims to create a collaborative movement dialog [38]. As the central training strategy in Capoeira, the game requires coordinated physical interaction between two participants and serves as the foundational communicative framework of the practice, functioning as a form of “physical dialog” between players [39]. Dyadic practice plays a central role in the learning process, fostering motor synchronization from the earliest training stages. This synchronization is reinforced through rhythmic music and the paired practice of symmetrical movements, commonly seen in beginner-level instruction [40]. Due to its emphasis on acrobatic exercises, weight-bearing positions, and movements inspired by gymnastics, practitioners of Capoeira often develop high levels of strength and flexibility [41]. Beyond the physical aspects, research indicates that Capoeira improves posture, motor coordination, and significantly contributes to emotional, social, and psychological development [42,43,44]. A recent literature review has shown that Capoeira engages a wide range of brain regions, including areas linked to motor, cognitive, executive, and emotional functions, suggesting its potential to enhance integrated neurocognitive processing [45]. A qualitative study in Israel explored the therapeutic benefits of teaching Capoeira to children, adolescents, and young adults in marginalized settings. Interviews revealed that the combination of movement, music, and social interaction provides a nonviolent outlet for aggression, enhances self-efficacy, and fosters a sense of belonging within a supportive community. These factors indicate that Capoeira may be an effective approach for social and emotional rehabilitation, particularly for those facing exclusion or rejection [46]. A qualitative study conducted in Brazil examined the role of Capoeira in promoting inclusion, enhancing self-esteem, and improving social interactions among children with ASD [47]. Utilizing interviews and observations at two therapeutic centers for children with special needs, the research revealed that participation in Capoeira led to significant improvements in motor skills, social participation, and self-efficacy. The children demonstrated increased confidence, greater initiative in social interactions, and enhanced coping mechanisms. Furthermore, the integration of movement, music, rhythm, and play provided a rich sensory experience that facilitated emotional and social engagement. These findings underscore Capoeira’s potential as an educational and rehabilitative space that supports the multidimensional development of children with ASD.

### 1.4. Capoeira and ASD in Israel

In Israel, it is estimated that approximately 10,000 school-aged children engage in Capoeira training across roughly 100 centers nationwide. Since the establishment of its activity over 35 years ago, the Israeli Capoeira community has developed comprehensive instructor training programs and routinely organizes professional conferences. During a national instructors’ conference held in January 2023, attendees were queried regarding the inclusion of children diagnosed with ASD within their elementary-age groups. All respondents affirmed the integration of children with ASD in their programs, spanning a range of functional levels. Furthermore, some groups provided support for children requiring ongoing mediation through the assistance of aides during training sessions. It is estimated that around 200 children aged 7 to 12 with ASD consistently participate in community-based Capoeira classes, which are conducted in the afternoons, across the country. Despite the increasing scholarly interest in the prospective benefits of Capoeira for individuals with unique developmental profiles [46], and the rising acknowledgment of its contributions to psychological resilience, self-esteem, and social bonding [48,49], there remains a lack of systematic research investigating the specific advantages of inclusive Capoeira groups composed of both children with ASD and their typically developing peers.

The significance of the study lies in presenting Capoeira as a socio-rehabilitative model that integrates motor, sensory, and communicative interventions within a mixed group framework that includes both children with and without disabilities. Such a setting fosters an environment that combines social inclusion with the promotion of various developmental skills.

A preliminary review of the existing literature reveals a notable lack of studies addressing the connection between Capoeira and ASD. Most existing works focus on the historical evolution of Capoeira or on its contribution to motor development among typically developing populations.

Although physical activity interventions such as swimming, horseback riding, and martial arts have demonstrated benefits for children with ASD, Capoeira remains virtually unexplored. Its distinctive blend of rhythm, movement dialog, and non-competitive interaction suggests potential developmental value. This qualitative pilot study aimed to examine parents’ perspectives on Capoeira’s potential contributions to their children’s motor, social, and sensory functioning, thereby providing preliminary insights to inform future research.

Beyond its theoretical contribution, this study also holds considerable practical potential. It may increase awareness among Capoeira instructors and trainers regarding the importance of including children with special needs in their programs, while also encouraging parents to consider enrolling their children in physically interactive activities that promote social engagement, emotional regulation, and a sense of belonging. In doing so, the study proposes an innovative framework for developing motor-social interventions grounded in rhythm, movement, play, and inclusion.

### 1.5. Research Aim

The current qualitative pilot study seeks to evaluate the suitability of existing measurement tools in preparation for a subsequent quantitative study that will examine the effectiveness of Capoeira training among children diagnosed with ASD. While established research-based instruments provide documented value, this study adopts a bottom-up, field-based approach aimed at exploring how participation in Capoeira is perceived to impact children’s motor and social functioning, as reported by their parents. As a pilot study, the findings are exploratory and intended to inform future quantitative research.

The research questions address the daily challenges parents, and their children, face due to ASD, the success of integrating children into mixed-ability training groups, the meaning parents attribute to their child’s participation, and the perceived motor and social changes resulting from the activity. Furthermore, the study investigates the instructional practices employed by Capoeira instructors, with the aim of identifying mechanisms and factors that contribute to successful group integration.

## 2. Materials and Methods

### 2.1. Procedure

This qualitative pilot study is based on semi-structured in-depth interviews with parents of children who had participated in Capoeira classes in Israel for at least one year. The study was approved by the university ethics committee (AU-HEA-LK-20240726). Following ethical approval, parents from across the country, whose children trained regularly with certified Capoeira instructors and who had voluntarily chosen to disclose their child’s ASD diagnosis, were invited to participate.

Recruitment was carried out via an invitation message distributed by the Capoeira instructors to parents whose children met the study criteria. Upon scheduling the interview, participants voluntarily agreed to take part in the study and signed an informed consent form.

### 2.2. Participants

A convenience sample was composed of ten parents (seven mothers and three fathers) of children with ASD, aged 7 to 15 (Mean age 10.05 ± SD = 2.63), who regularly participate in Capoeira classes in Israel. All children had been diagnosed with ASD during early childhood, and the diagnosis had been openly disclosed to the group instructor by the parents as described in Table 1. Eligibility required that participants be parents of children with a confirmed ASD diagnosis, who had trained in Capoeira for at least one year and had participated in Capoeira during their elementary school years (ages 7–12). Families that did not meet these conditions, were unavailable for an interview, or declined to participate were excluded. In total, 16 families were invited through Capoeira instructors; 10 met the inclusion criteria and agreed to participate, while 6 did not meet the criteria or declined participation. Regarding educational placement, seven children were enrolled in special education classes, two attended mainstream classrooms with the support of an aide, and one was integrated into a mainstream classroom without ongoing support. After nine interviews, a noticeable decline in the emergence of new information was observed, indicating data saturation. Following this observation, the research team made a joint decision to discontinue data collection following the tenth interview. Potential data interpretation bias was addressed through peer debriefing and iterative discussions within the research team. This approach was considered suitable given the exploratory and pilot nature of the study.

### 2.3. Research Instruments

Data was collected through in-depth interviews conducted by the first author (R.L.A). All interviews took place face-to-face and lasted between 20 and 50 min, with an average duration of approximately 35 min. The interviews were audio-recorded and transcribed with the participants’ consent.

The interview protocol began with an overview of the study and its objectives, along with relevant ethical information required by the institutional review board. Following this, the interviewer collected essential demographic data about the participating child, including age, duration of Capoeira participation, and training frequency. Subsequent questions focused on the primary research areas while allowing flexibility for participants to elaborate. The interview guide included topics related to the child’s background and diagnosis. Parents were initially asked to describe their child’s autism diagnosis and developmental challenges, with prompts such as, “Please describe your child’s autism; how does it manifest?” The conversation then shifted to the child’s engagement with Capoeira, addressing behavioral and emotional responses before and after training. Sample questions included: “How does your child behave prior to training?” and “What do they say about the class at home?” Additionally, the guide prompted parents to reflect on perceived developmental, emotional, or behavioral changes linked to Capoeira participation. Key questions included, “Have you noticed any changes in your child due to Capoeira? Can you describe their emotional state before and after a session?” To further explore, parents were invited to share their understanding of how Capoeira instruction may contribute to these changes. Lastly, the interview addressed the parent-instructor relationship and how the Capoeira environment supported inclusion. Questions included, “What would you say to a parent with concerns about inclusion? How has your experience with the instructor been?” Each interview concluded with expressions of gratitude for the parent’s participation and a brief note that a summary of the study’s findings would be shared upon publication.

### 2.4. Interview Analysis

The recorded interviews were transcribed verbatim and analyzed using a thematic approach, following an inductive process to identify core themes emerging from the participants’ narratives, including recurring language and patterns. Data analysis was conducted in accordance with established guidelines for thematic analysis [50].

Following an in-depth familiarization with the data, initial coding was performed manually by the first author, who repeatedly reviewed the transcripts and extracted illustrative quotations. The supervisory team (two senior researchers) subsequently reviewed the coding and transcripts, verified the appropriateness of quotations, and suggested refinements to the thematic structure. This was followed by the organization of participants’ perceptions and experiences into key themes, which were grouped into conceptual categories. After reviewing the themes and checking for consistency, each identified theme was clearly defined and supported by direct quotations from the interviews, selected to faithfully reflect the essence of participants’ views and the diversity of their perspectives. No qualitative data analysis software was used. The interviews were conducted in Hebrew only and there was no need for translation or re-translation during the data collection phase. In order to write the article in English, translation and scientific linguistic editing were performed accordingly.

### 2.5. Description of the Intervention—Participation in Capoeira Classes

The present study included parents of children diagnosed with ASD who regularly participate in Capoeira classes in cities across central Israel. The classes are led by certified Capoeira instructors licensed to teach, but do not have specialized training in working with children with special needs.

The autistic children are integrated into mixed-ability groups alongside typically developing peers of the same age. The majority of children represented in the sample (6 out of 10) train at least twice a week, with each session lasting a minimum of 45 min, and have been participating for over one year.

## 3. Results

This section is structured around three key themes that reflect the participants’ perceptions of how Capoeira training has influenced their children’s functioning. The main themes are the following:

(1) Common challenges associated with ASD;
(2) Experience of participating in Capoeira classes;
(3) Behavioral changes observed in the context of Capoeira.

Each overarching theme is further divided into sub themes that address specific topics highlighted by the interviewees, including: motor difficulties and sensory regulation; challenges in self-expression and social communication; preparation for and entry into training sessions; integration into mixed-ability groups; improvements in motor and sensory functioning; enhanced self-presentation; and increased understanding of others and social communication. All quotes included in this section are verbatim statements from the interviewees, enclosed in quotation marks and coded accordingly. Each code consists of a letter indicating the interview number and a numeral indicating the transcript page from which the quote was extracted, allowing for precise retrieval of the source material.

### 3.1. Common Challenges in ASD

This theme comprises two selected subthemes that address challenges commonly experienced by children with ASD, as reported by the interviewees. It focuses on difficulties in sensory regulation, motor functioning, alongside challenges in self-presentation and social communication.

#### 3.1.1. Difficulties in Sensory Regulation and Motor Functioning

This sub-theme reflects interviewees’ accounts of their children’s sensory and motor challenges and the impact of these difficulties on daily functioning. Many of the participating children were described as having experienced, either currently or in the past, difficulties with sensory regulation, spatial orientation, and basic motor skills. These challenges, particularly heightened sensitivity to stimuli such as noise or touch, often led to avoidance of social and physical activities and required careful planning of daily routines and community participation.

Interviewees described challenges in interpreting bodily sensations, such as atypical responses to pain: “He didn’t have a normal reaction to pain, he could get a bleeding open wound and just do nothing about it because he was busy with his own things” (A,2) and tactile sensitivities: “Hugs are impossible. He doesn’t like hugging, he dislikes physical contact” (J,3) as well as sensitivity to noise and crowded environments: “If there’s noise or lots of people, he wants to put on headphones and be alone” (B,4).

Such sensitivities often interfered with participation in social events and required families to carefully plan daily activities: “I really wanted to include him in the summer camp… but there were so many kids and such a range of ages, and it was very noisy… He just couldn’t manage, and after an hour, I had to take him home. Maybe next year” (A,6).

Interviewees also described vestibular and proprioceptive regulation difficulties, as well as balance issues: “He constantly spins in place and bumps into things” (A,2); “He keeps falling” (B,5). Interviewees noted their child’s motor challenges, especially when compared to peers of a similar age: “He had motor difficulties” (A,1); “At age three or four, he couldn’t hold things properly. He couldn’t grip a pencil the way he should” (I,5). Many of the children showed preference for sedentary activities and a general disinterest in physical activity: “He’s not a sporty kid. If he had a choice, he’d sit on the couch playing with Lego, watching TV, or drawing” (I,6).

Interviewees emphasized that one of the reasons they chose Capoeira was that it does not involve uncomfortable physical touch: “One of the things that fascinated me about Capoeira is that there’s no harsh or unpleasant touch” (A,6). Others emphasized previous difficulties with contact-based martial arts: “We tried judo, and he didn’t like being touched. I remember thinking: in Capoeira they don’t touch each other that much, maybe that’ll work for him” (H,3).

#### 3.1.2. Difficulties in Self-Presentation, Following Instructions, and Social Communication

This sub theme is directly related to the communication challenges often associated with ASD. Interviewees expressed concerns about these challenges and shared their desire to identify supportive strategies that could foster development in this area. Many interviewees indicated that recognizing these communication gaps prompted them to seek diagnostic evaluations and find activities that could promote social connection, openness to new situations, and motor development. Interviewees provided numerous examples of their children’s difficulties in responding to verbal cues or instructions. For instance: “He did not really follow instructions. He did not fully understand them, even though his cognition is completely intact, he did not always hear or was able to concentrate” (A,1); “He barely spoke, did not talk to strangers, and did not always respond to his name” (A,2); “He tends to ignore; he is in his own world” (D,2); “In daycare she was quiet. Very quiet, anxious, nodding” (E,1). Interviewees described avoidant or withdrawn behaviors linked to anxiety or frustration: “He would hide under the table when something upset him, he would shut down, become stressed, and refuse to cooperate” (I,1). Interviewees also described patterns of avoiding communication with other children: “He always plays alone besides the other children, and if someone tries to join him, he gets upset. If an unfamiliar person tries to talk to him, he does not engage” (B,2); “He is very quiet, plays alone, and you can barely notice him” (D,1); “The gap between him and the other children in class keeps growing, in interests and in how they communicate” (C,1); “He did not engage with other children at all. Especially with younger ones, he just ignored them” (F,11). Some interviewees spoke about a sense of alienation resulting from their children’s difficulties in developing effective social strategies: “Something is off about the way she communicates with her friends” (E,2); “He doesn’t understand, and then there are a lot of misunderstandings and people get angry at him” (V,3); “He really wants friends and to fit in, but it seems like he doesn’t know how”, “He does things that trigger rejection and get him the opposite result” (C,2). In light of these prevalent challenges among children with ASD, interviewees expressed a strong desire to find meaningful out-of-home activities that could support both motor and social development while also helping to reduce anxiety.

### 3.2. The Experience of Participating in Capoeira Classes

This theme explores the experiences of interviewees regarding their children’s participation in Capoeira classes and how these experiences contribute to their overall development and social integration. It includes two key subthemes: the process of preparing for and starting the activity, and the integration of children into mixed groups that include participants both with and without ASD.

An additional sub-theme not included here addresses the challenges of group integration and the supportive strategies implemented by instructors.

#### 3.2.1. Preparation for and Entry into the Activity

Transitions and shifts between types of activity can present significant challenges for children with ASD and their families [51]. This sub-theme focuses on the process of preparing for Capoeira lessons from the moment the child arrives from school, through dressing in training clothes and their behavior prior to the session, to the moment of entering the training space. It also describes how parents and instructors support children when difficulties arise during entry into and integration within the activity.

Interviewees observed unusual functioning in their children’s preparation for Capoeira, in contrast to the difficulties typically seen in other contexts. This was evident as early as school pickup: “When he knows it is for Capoeira, he is already waiting outside the classroom long before” (B,4); “When I pick him up from school, he shouts ‘Capoeira! already at the school gate’” (G,3).

Interviewees described a strong willingness among the children to prepare independently: “He comes home, says, ‘Dad, I have training today, I am going to get dressed, I am getting ready’” (J,7). They noted a clear contrast between the effort needed to prepare for other frameworks and the ease of preparation for Capoeira: “He comes home and asks me for the shirt and pants. It takes him one minute to get ready. So even though in the morning I have to dress him up, when it is for Capoeira, I do not need to do anything, not help at all,” (B,4).

This independence was also reflected in readiness for class: “He already gets dressed on his own, gets ready on his own, takes a bottle on his own, maybe that is normal for other kids, but for us, it is not” (F,8).

Interviewees described strong anticipation for the training session and eagerness to participate: “He is happy, he is waiting, he knows when the class is” (A,1); “He truly looks forward for the training, really wants to go to Capoeira” (D,4). Interviewees also described a strong desire in their children to begin the activity: “He literally runs from the house to Capoeira” (B,4).

Few interviewees described challenges in getting the child out of the house when they were immersed in another activity: “It takes a push because when he is watching TV, it is hard to get him out, but (when it comes for Capoeira) it is not very hard. He likes the classes, he likes going” (I,5).

Interviewees emphasized the importance of a consistent and predictable structure, noting that disruptions to the training routine could lead to resistance: “If they move the day, he will suddenly start to struggle and will not want to go” (H,3).

Preparation strategies, such as pre-session reminders and priming, were described as essential tools for easing transitions: “When is it hard to leave? When I do not remind her in time” (E,5).

Additional challenges related to adjusting to the instructional staff were raised, particularly when there was an unexpected change of instructors. While adapted responses, such as assigning a personal mentor, supported successful engagement: “He has a mentor who helps him with the exercises” (G,2), the absence of such support could disrupt the entry into the session: “If the mentor were late or did not show up, there would be crying” (G,2).

Interviewees shared various strategies to manage staff transitions, such as receiving a photo of the substitute instructor in advance: “If there is a substitute teacher, we will only come after that teacher sends me a photo of himself” (G,3).

Another approach to easing transitions was arriving early to build familiarity: “We come fifteen minutes early, and the instructor comes outside and introduces himself to the child. He says, ‘Do you want to be my assistant?’ Then we go in fifteen minutes early to lay the groundwork” (G,3).

In general, interviewees described entry into the activity as a positive experience for their children: “I see that when he enters Capoeira, he suddenly feels calm” (G,2), and highlighted the independence their children demonstrated: “I arrive, he says hello, goes in, takes off his shoes, grabs his bottle, goodbye” (G,2); “He also wants to be independent, so when we arrive at the gym he tells me: ‘I am going up by myself, do not come with me’” (D,4).

These findings suggest that participation in Capoeira elicits a unique sense of motivation in interviewees’ children, reflected in exceptional cooperation during preparation, demonstrated independence when entering the session, and the ability to engage without ongoing support. Unlike other activities, Capoeira training was perceived as stimulating interest and commitment, serving as an opportunity to develop life skills and independence. However, alongside this enthusiasm and self-initiated participation, unexpected changes, lack of preparation, or disruptions to routine could hinder the child’s ability to leave the house or participate effectively. Interviewees stressed the importance of proactive strategies such as verbal or visual priming, maintaining a consistent framework, and establishing familiarity with the instructional staff to support successful participation.

#### 3.2.2. Integration into a Mixed Group

Programs designed for children with ASD typically focus on therapeutic or one-on-one settings, such as therapeutic horseback riding, hydrotherapy, speech therapy, or physiotherapy. While dedicated group settings allow for peer interaction among children with similar characteristics, research has shown that integrated groups, including children with typical development alongside those with ASD can significantly support the acquisition of life skills and communication through peer-mediated learning [7,52]. This subtheme presents the perspectives of interviewees regarding the challenges their children and instructors face when integrating children with ASD into mixed groups, as well as their impressions of the social environment in Capoeira classes. It also explores their views on their children’s social experiences and how the core principles of Capoeira aid in successful inclusion. Interviewees emphasized the inclusive and nonjudgmental atmosphere of the group, where their children receive equal treatment, experience social successes, and develop a sense of belonging and self-expression. Capoeira’s unique qualities, especially its focus on cooperation rather than competition, help create a social environment that enables children with ASD to actively participate, achieve success, and form friendships with their peers. These findings highlight the significant role of integrated settings in fostering belonging and helping children develop social skills within an inclusive environment.

Interviewees expressed a desire for their children to participate in a group-based, inclusive environment. One parent noted, “Group sports create social connections and a social circle, and that is what my child has been missing all his life” (F,12). However, they also shared concerns about integration in such settings. One interviewee remarked, “I see the children he is with, these are not children on the spectrum” (G,2), while another expressed worries about potential conflicts: “I was worried. I told the instructor: ‘What if he starts fighting with the other kids? What if something does not work and he loses control? It will be hard for him’” (J,1). Some interviewees recognized the emotional challenge of placing their child in a group that differed from them: “To bring a child on the spectrum into a group of children who are not on the spectrum is already a challenge for the child” (J,8). From the interviews, it emerges that Capoeira classes are seen as socially inclusive environments. As one parent described, “He might start doing hand movements or dancing, things that typical kids do not usually do. Yes, they are very understanding, these kids” (J,8). Another parent shared, “It is his safe space, a place where they understand him, where being different is accepted. He does not feel different when he is there; that is one of his happiest things. The other kids say hello to him, he feels like one of the group” (G,4). Another noted, “They form a circle, and he steps in proudly and receives encouragement” (H,5). Interviewees also highlighted that this environment allows their children to learn appropriate behaviors from peers. One parent explained, “He tells me he can ask that kid or some of the others, if he is in a situation he does not understand. They will stop him and say, ‘That is not appropriate’, and he accepts it well” (F,3).

Capoeira is not a competitive sport; training for elementary-aged children emphasizes cooperation within the game. Interviewees viewed this aspect as a significant reason why Capoeira meets their children’s needs, distinguishing it from other activities that are competitive: “There is no element of competition. You do your best relative to yourself.” (E,8). Another said, “First of all, because it is not competitive, it is more of a social sport” (H,5). One parent remarked, “What I think works great for my child specifically is that it is not competitive. The goal is for everyone to succeed” (F,2). Another added, “I do not want him doing any competitive sport. Nothing. That would just make his anxiety worse. He had meltdowns when he lost at chess, he would be upset for days” (H,10). In Capoeira, however, the atmosphere is positive and supportive: “Here it is the opposite. If someone pulls off a move, ‘Wow! That was awesome! How did he do that?’ There is so much positive feedback, and it has always been that way” (F,12). Interviewees frequently described the sense of acceptance their children experience in Capoeira: “At some point, the girl from home also became the Capoeira girl. That means she feels very comfortable” (E,7). A parent shared, “My son is a bit reserved about his Autism, but there, it is like he came out of the closet. He does not hide it. The day he got the diagnosis, he walked into the studio and said, ‘Hey, I am Asperger’s. I am autistic…’” (F,4). Another parent noted, “I think it is the place where he feels free to express himself, to release energy and enjoy himself” (G,5). One interviewee reflected on how the social atmosphere of the group carried over beyond the class itself: “When it is our turn to drive them home, they laugh, there is this social vibe, something that usually does not happen at school. It really gives him a place to feel comfortable being himself, because he does not feel that way in most places in the world” (F,15). Interviewees indicated that Capoeira has become a meaningful social framework for their children: “Capoeira is her world, she is a part of it” (E,5). Another parent expressed, “Of course he loves it. It is like he found his place” (C,3). The parents emphasized the importance of having frameworks where their children can integrate without feeling excluded: “It means a lot because he is not supposed to feel different there. He already feels different in other areas, such as going to the park, playing with kids, or joining other activities, he needs to feel like everyone else” (G,9). These findings suggest that for the interviewees’ children, Capoeira is not viewed merely as a physical activity but as a meaningful social space that allows for natural inclusion in a mixed group. As one parent put it, “In Capoeira he knows how to be with those who are better than him and those who are less good than him, without making a big deal of it, like he usually would in other situations” (C,6).

### 3.3. Changes Related to Being Involved in Capoeira

This theme presents interviewees’ accounts of changes they observed in their children following participation in Capoeira classes, with a focus on sensory-motor functioning and social skills. It includes two selected sub-themes: improvement in sensory regulation, motor functioning, and enhancement of self-presentation and social communication.

#### 3.3.1. Improvement in Sensory Regulation and Motor Functioning

This sub-theme presents the interviewees’ observations of changes in their children following participation in Capoeira classes, specifically regarding their ability to cope with external stimuli, regulate sensory input, perceive their bodies in space, and improve motor development. According to the interviewees, Capoeira had a positive impact on their children’s sensory regulation, evident in their improved responses to external stimuli, increased bodily awareness, and heightened calmness after training sessions. The music in Capoeira classes was viewed as a significant factor in enhancing auditory regulation and fostering a sense of social belonging.

Interviewees considered Capoeira a major contributor to their children’s motor development, noting reduced clumsiness and notable improvements in coordination, balance, and overall movement abilities. In addition to physical progress, they emphasized that Capoeira expanded their children’s movement repertoire and enhanced their sense of competence.

Most interviewees highlighted the importance of movement in responding to their children’s need for physical stimulation, with one stating: “Of course, it gives him a lot in terms of sensory input. It is really important, he is a child who needs much physical stimulation, and the fitness and physical activity affect him very positively in every aspect, including his nervous system” (C,5). They described improvements in handling sensory challenges, such as wearing clothing: “In the last two times, he made an extra effort to come in long pants, which is totally uncharacteristic for him” (C,4).

Further improvement was observed in the children’s ability to perceive their body’s position in space and to act accordingly: “They feel their bodies better” (B,7) and “He really improved at that. For example, when he wants to do a cartwheel, in the past, if he tried, he would hit someone. Now he looks around, moves people out of the way, makes sure it is clear, and only then does it” (D,6)

Interviewees spoke about the unique role of music in supporting their children’s auditory regulation. While loud environments are generally perceived as stressful or overwhelming, the music in Capoeira sessions was experienced differently: “There are Capoeira songs he really likes” (G,2). One parent noted, “Usually, when there is noise, lots of things, people, he wants to wear headphones and be alone. Moreover, he usually stands at the side. That is what happened when we got there at first. But as soon as the music started, he took off the headphones and went in” (B,4). Another shared, “I can go with him to a family event, and he cannot stand the noise. But here, he is okay with the noise” (H, 6). It seems that the simple musical patterns played by participants themselves created a unique sense of belonging: “When they all stand and play music together, he really feels part of something, something he feels equal in. He feels like he is in rhythm with everyone. He does not experience that in other places” (C,4). Interviewees emphasized the significant motor improvements their children experienced through Capoeira, particularly given the motor challenges typically associated with ASD: “For my child, who is clumsy and not very physically active, it is great. It is exactly what he needs” (I,8); “His motor skills and physical abilities improved” (D,6) and “We saw his coordination improve. We noticed his body becoming smarter, with fewer injuries and bumping into things less, even when spinning” (A,3).

These improvements were evident in everyday functional tasks as well: “Now when he runs, he does not fall. I can see him doing things in a much more organized way” (B,5). Beyond physical improvement, interviewees reported that Capoeira greatly enhanced their children’s confidence and sense of capability: “He enjoys it, he gets really excited if he suddenly manages to do a cartwheel” (A,4); “It gives him confidence and something to be proud of” (D,6). Additionally, Capoeira enabled children to engage in other movement-based activities: “It suddenly opened us up to a whole new world of body control that just did not exist before” (A,3); “This year, I signed him up for hip hop, and I am sure he would not have been able to do that if he had not gone through a year of Capoeira” (B,5). Interviewees also noted a sense of calmness in their children after Capoeira sessions: “When he comes out of there, it is like he took a breath” (B,5); “She comes back much more relaxed” (E,8).

#### 3.3.2. Improvement in Self-Presentation, Understanding Others, and Social Communication

This sub-theme discusses the interviewees’ reflections on the changes they observed in their children’s self-presentation and communication skills after participating in Capoeira. The findings suggest that interviewees view Capoeira as an impactful tool that has significantly enhanced their children’s social communication abilities, allowing them to present themselves positively and confidently to others. Through their membership in an inclusive community, showcasing personal skills, and engaging in teaching roles, children developed greater self-confidence and improved their ability to initiate social interactions. The movement-based interactions at the core of Capoeira were described as fostering awareness of others and enhancing adaptive communication skills. Interviewees highlighted Capoeira’s contribution to developing cognitive flexibility and adaptability in dynamic social situations, noting that the skills learned in Capoeira present a carry-over and extend beyond the class applying to broader social contexts.

Interviewees expressed that Capoeira became a significant aspect of their children’s identities: “Whenever someone asks him what he does in the afternoon, he says, ‘I do Capoeira’” (H,3). They described how Capoeira’s visual symbols, like the training uniform, act as tools for connection: “If he sees someone wearing a Capoeira shirt outside, he’ll go straight over to talk to him, saying, ‘I do Capoeira too; I play it too’” (C,4). Interviewees reported that their children use Capoeira skills to connect with others: “When we are in an open place with other people, he almost always demonstrates Capoeira moves, it gives him something positive to engage with and to present himself through” (C,5). They also noted increased social openness and a greater ability to engage with others in everyday situations: “I see he is more open, if someone talks to him, he does not shut down” (B,6). This improvement was linked to a rise in the children’s self-confidence: “His confidence has significantly improved, he used to be very shy. Now he approaches other kids without any problem at all” (D,6).

Activities typical to Capoeira were experienced as supportive in understanding others’ feelings: “There was a strong emphasis on whether your partner is ready, making eye contact. He knows how to look and wait to see if the other person is ready. That he can ask, ‘Are you ready?’” (A,3); “In Capoeira, he has the opportunity to invite another person to practice or play, to understand boundaries, knowing when it is appropriate. You are doing something that reflects onto the other person, and you have to anticipate their movements while allowing them to anticipate yours. You are across from them, but not against them. It strengthens reciprocity, which he, as a child with ASD, really struggles with” (C,7).

The mutuality embedded in Capoeira was described as a catalyst for developing essential skills for communicative interaction: “There is a kind of dialog in Capoeira with the person you are with” (D,5); “It is like a type of interaction, only more movement-based” (I,9); “The whole structure of Capoeira, I feel like it gives him more cognitive flexibility. He understands that he has to go with the flow of the other person, for example, he has to duck so the kick passes over him. I actually see it as a metaphor for real-world relationships and interpersonal dynamics” (F,8).

Interviewees also shared how their children used Capoeira experiences to support others: “His brother was terrified, and he used Capoeira to show him how you train, try, try again, until you succeed, until you overcome the fear” (C,5). They expressed their impression of improvements in their children’s behavior toward others: “I see how he acts toward others and how he eats, he has become more patient with other kids” (F,15). Interviewees spoke of their children’s growing desire to share and teach what they have learned: “Sometimes he even offers to teach a little to younger kids” (C,5); “She likes to talk, demonstrate, and explain things to kids, she enjoys teaching” (E,6).

Opportunities to take on instructional roles were described as empowering moments: “The instructor uses her sometimes to demonstrate, and she feels responsible and committed, it boosts her confidence” (E,6). They emphasized the importance of the skills developed through instructional experience: “He was given the responsibility of being an assistant instructor, and that took him one step forward. Until then, he did not even know how to interact with little kids” (F,10).

### 3.4. Summary of the Findings

Through interviews with parents, four primary domains of functioning were identified in which their children exhibited notable changes following participation in Capoeira: motor functioning, sensory regulation, social communication, and sense of belonging. Parents reported advancements in their children’s ability to manage external stimuli, enhanced bodily awareness concerning spatial orientation, decreased motor clumsiness, and improvements in coordination, balance, and overall movement capabilities. The incorporation of music within Capoeira was recognized as a significant factor in facilitating auditory regulation and strengthening the children’s sense of social belonging.

In the area of social communication, parents observed improvements in their children’s ability to initiate interactions, elevated self-confidence, and heightened awareness of others’ emotional and social cues. Parents described Capoeira as a practice that cultivates cognitive flexibility and adaptability to dynamic social contexts, with the social skills acquired during training having a carryover effect, being applied outside of the class environment. Additionally, participation in Capoeira was noted to inspire a distinctive sense of motivation in the children. This was evident in their cooperation during preparation, their demonstration of independence upon entering class, and their ability to engage in the activity without requiring continuous support.

Another significant factor identified was the supportive social environment within the Capoeira group, which naturally facilitated the children’s integration into a mixed setting. The inclusive atmosphere and equitable treatment contributed to positive social experiences, reinforced a sense of belonging, and encouraged the development of peer relationships, emphasizing cooperation over competition.

## 4. Discussion

The current study set out to explore the effect of Capoeira training on children with ASD. This qualitative pilot study is based on ten in-depth interviews with parents of children with ASD who participate in Capoeira classes. The aim was to explore the contributions of Capoeira across various domains, including motor and physical benefits, social skills development, experiences in mixed training groups, influences on executive functions, due to the activity. The significance of this study lies in its contribution to the limited understanding of Capoeira as a movement-based and social intervention for children with ASD, a topic that remains underexplored in the research literature. The findings suggest that Capoeira may positively impact all examined domains, with the most significant improvements observed in motor and social skills. Emerging evidence indicates that interventions focused on motor functioning may have broader positive effects on core symptoms of ASD, including social interaction, information processing, and executive functions [14,15]. According to parents’ reports, the findings of this study support this assertion by presenting evidence of enhancements in movement abilities, sensory regulation, cognitive flexibility, functional independence, motivation, social communication, and social initiative. These results highlight Capoeira’s potential as an integrated model that combines physical activity and social intervention.

From a motor perspective, interviewees reported that their children showed improvements in coordination, spatial body awareness, balance, and a reduction in falls. Additionally, they observed increased physical strength and a broader range of movement. These findings are consistent with a previous study [53], which highlighted the significant contribution of martial arts to the development of motor skills among children with ASD. The results suggest that participation in Capoeira enhanced the children’s awareness of their body position in space, contributing to improved movement control, collision avoidance, sensory regulation, and the ability to perform movements in greater synchrony with the surrounding environment. Interviewees noted a significant reduction in motor clumsiness, including fewer collisions with objects and fewer falls and injuries. They also described greater bodily awareness, such as being mindful of their surroundings before attempting a cartwheel.

One of the distinctive aspects of Capoeira is its emphasis on reciprocal movement-based interactions, such as movement imitation and partner-based activity. These interactions require spatial awareness and promote motor synchronization. In Capoeira, children who struggle with spatial perception or motor coordination had the opportunity to explore adapted execution strategies while being encouraged to cooperate with their peers.

Additionally, Capoeira was found to expand the children’s movement repertoire, thereby not only supporting motor functioning but also contributing to developing body awareness and a sense of competence. Interviewees described improvements in self-esteem, feelings of pride, and self-confidence, emphasizing the unique nature of Capoeira in offering a non-competitive opportunity for success based on personal progress.

In the domain of sensory regulation, the study points to the contribution of Capoeira participation in improving children’s sensory self-regulation. This was reflected in better responses to external stimuli, increased body sensory awareness, and better management of internal tension. Movement during Capoeira training served as a regulatory tool, particularly for children with heightened needs for physical stimulation. Interviewees reported a heightened sense of calm following Capoeira sessions and described the training as a means for their children to release tension and regulate the elevated levels of physiological arousal often characteristic of some children with ASD. Similar sensory improvements following physical activity interventions have also been documented in other studies [54,55], especially when the intervention involved vestibular-related movements similar to those practiced in Capoeira [56].

Furthermore, the findings indicate that the music used during Capoeira sessions contributed to the children’s sensory regulation, particularly among those with heightened sensitivity to noise. This outcome aligns with previous findings demonstrating that music can support sensory regulation and reduce auditory overload in children with ASD [57,58]. Moreover, music played a central role in strengthening the children’s sense of social belonging. This effect is further supported by studies showing that participation in musical activities helps foster social bonding and a sense of inclusion within a group [59,60].

A common characteristic observed in children with ASD is difficulty transitioning between activities [2] and challenges in preparing for these activities [61]. The findings of the current study revealed a notable contrast between the difficulties these children faced when preparing for various activities and their high motivation and exceptional independence when preparing for Capoeira training. They also exhibited positive anticipation regarding their attendance and participation, which seems to be uniquely associated with Capoeira. Entry into the activity was found to be a significant phase in the integration process, during which the children showed initiative and autonomy, with little to no need for ongoing support. It appears that the structured and consistent framework of the training sessions provided a sense of security, reducing resistance to change. Specifically, priming, schedule consistency, and familiarity with instructors played vital roles in easing their transition into the activity.

In the social domain, the study highlights the unique contribution of Capoeira to developing social skills and a sense of belonging. Unlike most existing interventions, typically in one-on-one settings where the child mainly interacts with a therapist, Capoeira provides a natural group framework for social skills practice within a structured and supportive environment. Integration into a mixed group enabled the children to practice communicative skills while forming organic social connections with peers within the Capoeira sessions, as well as extending their social skills to social situations unrelated to the Capoeira training (in family gatherings and with small children). These findings reinforce previous research demonstrating that participation in integrated group settings contributes to acquiring life skills and improving social functioning of children with ASD [10,62].

An inclusive, accepting social climate was identified as a key factor in successful integration. Interviewees emphasized that the Capoeira environment was characterized by social acceptance and encouragement, which allowed the children to feel part of the group without being seen as different. This setting provided an opportunity to practice and improve social skills in real time while learning appropriate behaviors and understanding social norms. Compared to special education settings, where group sizes tend to be smaller and more structured, participation in larger and more diverse groups contributed to the children’s ability to adapt to dynamic social environments outside the Capoeira setting and within daily situations.

The practice of Capoeira, commonly known as “the game,” focuses on reciprocal movement interactions and has been shown to enhance nonverbal communication skills. Activities such as movement imitation, maintaining a shared rhythm, and coordinating with a partner enhanced the ability of children with ASD to interpret and respond to social cues while fostering awareness of others’ emotional and physical states. Additionally, the absence of competition emerged as a significant advantage. While many sports activities focus on achievement and winning, Capoeira encourages cooperation, mutual empowerment, and personal success. The children learned to celebrate the achievements of others and to enjoy the process itself without experiencing the pressure or frustration often associated with losing. Interviewees noted that this characteristic reduced social anxiety and improved the children’s ability to integrate into the group without fear of failure.

In addition to enhancing their ability to form social connections, Capoeira empowered the children to initiate interactions within the Capoeira setting but also beyond their Capoeira group and into daily situations. The children identified themselves as “Capoeira practitioners,” using the skills they had acquired to present themselves to others and establish points of connection with their environment. Actions such as demonstrating movements for peers, assisting younger children, and taking on instructional roles within the Capoeira setting strengthened their self-confidence and ability to engage in social situations.

The findings indicate that Capoeira served as a supportive framework, allowing children to develop social skills in an inclusive and nonjudgmental environment. The combination of cooperative movement activities, group support, and the absence of competition created a safe space for forming relationships, practicing communication, and strengthening the sense of social belonging.

## 5. Conclusions and Recommendations

The findings of the present study underscore the potential and perceived contribution of Capoeira as an integrated model of physical, behavioral, sensory and social intervention for children with ASD. Parents reported that Capoeira was perceived to enhance motor skills, sensory regulation, social skills, ease of transitions, self-confidence, and a sense of belonging. It offers a unique framework that is non-competitive and instead based on cooperation and mutual empowerment.

Capoeira was described as providing motor benefits through perceived improvements in coordination, balance, and proprioceptive awareness, and a reduction in motor clumsiness. In the social domain, Capoeira was reported to provide an inclusive group environment that enabled the practice of nonverbal communication, social initiative, and adaptability to changing situations, while reinforcing the children’s sense of belonging and self-worth.

These findings align with emerging evidence in the research literature, pointing to a possible link between improvements in motor functioning and gains in cognitive and social domains among children with ASD. They reinforce the understanding that motor intervention should not be viewed as an isolated goal but may contribute to broader functional outcomes. Given this understanding, it is advisable to explore the integration of Capoeira into educational and therapeutic interventions for children with ASD, especially within inclusive group settings that promote full participation with peers and in the broader community.

To enhance the success of inclusion in Capoeira specifically and in leisure activities more broadly, it is recommended that comprehensive professional training for Capoeira instructors in the field of Autism be provided, support systems tailored to the individual needs of each participant developed, and parents involved as active partners in the process. In addition, future research should examine the long-term effects of the intervention and identify the specific mechanisms that contribute to its success.

The findings of the current research suggest that in future studies regarding Capoeira and children with ASD, valid and objective outcome measures regarding individual goals (such as the goal attainment scale—GAS), sensory elements (such as the Short Sensory Profile—SSP), motor elements (such as the Movement Assessment Battery for Children—Movement ABC) and Autism severity (such as the Childhood Autism Rating Scale—CARS) should be used.

## 6. Limitations

The findings of this pilot study are based on interviews with parents of children with ASD, reflecting their perceptions through self-reporting. The goal of this study was to explore the full potential of content that may be beneficial for individuals with autism. However, as a qualitative pilot study, it does not permit causal inferences and given the small sample size and absence of a control group, its external validity remains limited. Therefore, it cannot be determined with certainty that the reported changes resulted directly from participation in Capoeira, as they may also be influenced by other factors, such as the child’s age, their position on the Autism spectrum, or natural developmental progression.

Additionally, since no objective measures were used, and no comparison was made with a control group, this study cannot determine whether functional progress occurred specifically due to Capoeira. Recruitment via Capoeira instructors may also have introduced selection bias, as families with more positive attitudes toward Capoeira or stronger connections to the instructors may have been more likely to participate. As an exploratory pilot study, the conclusions should be understood as exploratory, pointing to possible directions for further investigation. Future quantitative research, including a control group and precise measurement tools, is essential to assess the actual contribution of Capoeira to improvements across various domains. Due to these limitations, further quantitative research is warranted to allow for the control of intervening variables such as those identified here. The study intends to pursue these directions in future investigations, after the benefits of capoeira for autism are detailed in this qualitative study.

## Figures and Tables

**Table 1:** Socio-demographic and Capoeira participation details of children with ASD.

#	Child’s Age (Years Months)	Sex	Age at Diagnosis	Autism Level	Age Started Capoeira	Capoeira Duration (Years)	Training Intensity (Recent)
1.	7.6	M	3	Mild- Moderate	4	3.5	1
2.	7	M	3	Mild- Moderate	6	1	1
3.	12	M	10	Mild	8	4	2
4.	9	M	3.5	Mild- Moderate	7	2	1
5.	10	F	9	Mild	5	4	2
6.	15	M	11.5	Mild	5	10	3
7.	10.7	M	2.5	Low Functioning	7	3.5	1
8.	13	M	3.9	Mild- Moderate	7	6	2
9.	9	M	8	Mild	4	5	2
10.	7.5	M	4	Mild	6	1.5	2
Mean ± (SD)	10.0 ± (2.6)		5.8 ± (3.4)		5.9 ± (1.3)	4.0 ± (2.6)	1.7 ± (0.6)

Note: Training intensity (recent) refers to the average weekly frequency of Capoeira sessions during the last year.

## Data Availability

Due to the sensitive nature of the qualitative interviews and the need to preserve participant confidentiality, the data (full transcripts) are not publicly available. Summarized findings are presented in the article.

## Abbreviations

The following abbreviations are used in this manuscript:

ASD	Autism Spectrum Disorder
TD	Typically Developing
SD	Standard Deviation
GAS	Goal attainment scale
SSP	Short Sensory Profile
Movement ABC	Movement Assessment Battery for Children
CARS	Childhood Autism Rating Scale
